# The complete mitochondrial genome of *Labidocera rotunda* Mori, 1929 (Copepoda: Calanoida) from Jeju Island, Korea

**DOI:** 10.1080/23802359.2022.2122748

**Published:** 2022-09-23

**Authors:** Jimoon Jun, Hyeon Gyeong Jeong, Hyeongwoo Choi, Hyunmin Woo, Donggu Jeon, Young-Jin Seo, Seong-il Eyun

**Affiliations:** a Department of Life Science, Chung-Ang University, Seoul, Korea; b Department of Taxonomy and Systematics, National Marine Biodiversity Institute of Korea, Seocheon, Korea

**Keywords:** *Labidocera rotunda*, mitochondrial genome, copepod, calanoid, Pontellidae

## Abstract

We sequenced the complete mitochondrial genome of the copepod *Labidocera rotunda* (family Pontellidae) collected from Ihotaewoo Beach in Jeju, Korea. The mitochondrial genome was 16,564 bp in length and contained 13 protein-coding genes (PCGs), 22 transfer RNAs, and two ribosomal RNAs. The concatenated phylogenetic tree of *L. rotunda* was reconstructed using the maximum-likelihood method based on the eight PCGs obtained from eight species of copepods including *L. rotunda*. The results of the phylogeny analysis showed that *L. rotunda* was closely related to the family Temoridae among the three families. The complete mitochondrial genome of *L. rotunda* analyzed for the first time in this study provides insight into the phylogenetic and evolutionary relationship of *Labidocera*.

The genus *Labidocera* Lubbock, 1853 occurs mostly in the surface waters (0–30 cm surface layer) of the warm temperate to tropical regions along the Indian Ocean and the Pacific Ocean (Fleminger 1967; Razouls et al. 2005–2022). To date, a total of 63 species are included in the genus *Labidocera* (Walter and Boxshall 2021) and new species continue to be added (Eyun 2017; Mulyadi 2021). With species-specific sexually modified anatomical features and relatively large size among the copepod, *Labidocera* has been a notable subject for examination of the ecological, distributional, and morphological relationships between closely related species (Fleminger 1967; Jeong et al. 2014). Despite its advantages in research, the mitochondrial genomes (mitogenomes) of *Labidocera* are yet to be made available. Therefore, we conducted the complete mitochondrial sequencing of *L. rotunda*.

We collected *L. rotunda* from Ihotaewoo Beach in Jeju Island, South Korea (33°31′51.3″N, 126°25′15.4″E and 33°31′54.5″N, 126°25′33.6″E) using a conical net (mesh size 200 µm; mouth diameter 60 cm). Note that this site is not a protected area by the government and no permit was required. The voucher specimens were reexamined and cataloged at the National Marine Biodiversity Institute of Korea (https://www.mabik.re.kr/html/kr/, Hyeon Gyeong Jeong, hgjeong@mabik.re.kr) under the voucher number (MABIK CR00123334). Long and accurate PCR was performed to amplify the complete mitochondrial genome sequence using a set of universal primers (Wan et al. 2012) and designed primers. Sequencing was conducted using the primer walking method on an ABI 3730XL DNA Analyzer (Applied Biosystems, Inc., CA). Then, mitogenome annotation was initially conducted using the webservers, MITOS (Bernt et al. 2013) and MITOS2 (Donath et al. 2019), and manually modified based on the related species. The complete mitogenome of *L. rotunda* (NCBI accession number ON332184) is 16,564 bp in length, which is similar to the reported calanoid species (Eyun et al. 2007, 2017; Zhang et al. 2020). The mitogenome contains 37 genes comprising a conserved set of 13 protein-coding genes (PCGs), two ribosomal RNA genes (12S and 16S), and 22 transfer RNA genes. The overall base composition of the mitogenome is estimated to be 31.6% for A, 36.6% for T, 14.8% for C, and 16.9% for G, with a [A + T] content of 68.3%, which is similar to that of *Phyllodiaptomus praedictus praedictus* (family Diaptomidae) (69.4%) (Zhang et al. 2020). The concatenated phylogenetic tree of *L. rotunda* was reconstructed using the maximum-likelihood method with the substitution model of mtZOA + F+G4. Bootstrap analysis was performed based on the eight PCGs with 1,000 replicates to evaluate the confidence level of each clade (Figure 1). *Tigriopus californicus* (order Harpacticoida) was used as the outgroup in the phylogenetic tree. The well-supported bootstrap value of 100% indicates the close relationship between *L. rotunda* (family Pontellidae) and *Eurytemora affinis* (family Temoridae).

**Figure 1.**
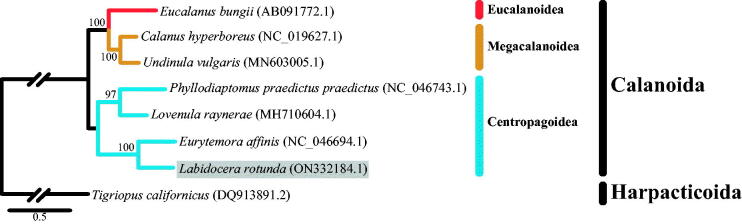
Maximum-likelihood (ML) phylogeny of eight copepod species (seven from Calanoida including *Labidocera rotunda* and one species of Harpacticoida) based on the eight concatenated protein-coding genes of amino acid sequence. *T. californicus* (order Harpacticoida) was used as the outgroup. The numbers on the branches indicate ML bootstrap percentages (1,000 replicates). The GenBank and NCBI accession numbers for the published sequences are incorporated. The gray box indicates the *L. rotunda* analyzed in this study.

This study is the first to present the complete mitochondrial genome of *L. rotunda*. The results obtained here provide insights into the phylogenetic and evolutionary relationship of *Labidocera*.

## Data Availability

The mitochondrial sequence is available in NCBI’s GenBank under the accession number: ON332184 (https://www.ncbi.nlm.nih.gov/nuccore/ON332184.1).
